# Ubiquitin carboxyl-terminal hydrolase 1 (UCHL1) is a potential tumour suppressor in prostate cancer and is frequently silenced by promoter methylation

**DOI:** 10.1186/1476-4598-10-129

**Published:** 2011-10-14

**Authors:** Ramesh Ummanni, Edgar Jost, Melanie Braig, Frithjof Lohmann, Frederike Mundt, Christine Barett, Thorsten Schlomm, Guido Sauter, Tina Senff, Carsten Bokemeyer, Holger Sültmann, Catherine Meyer-Schwesinger, Tim H Brümmendorf, Stefan Balabanov

**Affiliations:** 1 Department of Oncology, Haematology and Bone marrow transplantation with section Pneumology, Hubertus Wald-Tumour Zentrum (UCCH), University Hospital Eppendorf (UKE), Hamburg, Germany; 2 Klinik für Onkologie, Hämatologie und Stammzelltransplantation, RWTH Aachen University, Aachen, Germany; 3 Department of Internal Medicine, Nephrology, University Hospital Hamburg-Eppendorf (UKE), Hamburg, Germany; 4 Martini-Clinic, Prostate Cancer Center, University Hospital Eppendorf (UKE), Hamburg, Germany; 5 Department of Pathology, University Hospital Eppendorf (UKE), Hamburg, Germany; 6 Cancer Genome Research, Deutsches Krebsforschungszentrum (DKFZ), Heidelberg, Germany

**Keywords:** prostate cancer, UCHL1, ubiquitin system, tumour suppression, signalling

## Abstract

**Background:**

We have previously reported significant downregulation of ubiquitin carboxyl-terminal hydrolase 1 (UCHL1) in prostate cancer (PCa) compared to the surrounding benign tissue. UCHL1 plays an important role in ubiquitin system and different cellular processes such as cell proliferation and differentiation. We now show that the underlying mechanism of UCHL1 downregulation in PCa is linked to its promoter hypermethylation. Furthermore, we present evidences that UCHL1 expression can affect the behavior of prostate cancer cells in different ways.

**Results:**

Methylation specific PCR analysis results showed a highly methylated promoter region for UCHL1 in 90% (18/20) of tumor tissue compared to 15% (3/20) of normal tissues from PCa patients. Pyrosequencing results confirmed a mean methylation of 41.4% in PCa whereas only 8.6% in normal tissues. To conduct functional analysis of UCHL1 in PCa, UCHL1 is overexpressed in LNCaP cells whose UCHL1 expression is normally suppressed by promoter methylation and found that UCHL1 has the ability to decrease the rate of cell proliferation and suppresses anchorage-independent growth of these cells. In further analysis, we found evidence that exogenous expression of UCHL1 suppress LNCaP cells growth probably via p53-mediated inhibition of Akt/PKB phosphorylation and also via accumulation of p27kip1 a cyclin dependant kinase inhibitor of cell cycle regulating proteins. Notably, we also observed that exogenous expression of UCHL1 induced a senescent phenotype that was detected by using the SA-ß-gal assay and might be due to increased p14ARF, p53, p27kip1 and decreased MDM2.

**Conclusion:**

From these results, we propose that UCHL1 downregulation via promoter hypermethylation plays an important role in various molecular aspects of PCa biology, such as morphological diversification and regulation of proliferation.

## 1 Background

Prostate cancer (PCa) is the most common type of cancer found in men and is among the leading causes of cancer death in the western world [1]. The specific causes of prostate cancer remain poorly understood [2]. Recently, our group identified differentially expressed proteins which are significantly deregulated in PCa predicting their role in initiation and progression of PCa [3]. Among those proteins several members of the ubiquitin system have shown an altered expression. Ubiquitination of proteins has emerged as one of the most versatile post-translational modifications, regulating a diverse arrays of cellular processes [4]. Ubiquitination plays a central role in degradation of proteins both through proteasomal targeting and by lysosomal degradation. In recent years, it became clear that deubiquitination is a crucial process in multiple intracellular signaling pathways, resulting in putative oncogenic or tumor suppressive functions [5]. Deubiquitination of proteins is catalyzed by a set of enzymes known as deubiquitinases (DUBs). In the human genome approximately one hundred human DUBs are known so far classified into five categories: ubiquitin specific proteases (USP), ubiquitin C-terminal hydrolases (UCH), ovarian tumour proteases (OTU), Josephins and the Jab1/MPN/MOV34 metalloenzymes [5,6].

Ubiquitin C-terminal hydrolase L1 (UCHL1), a member of the UCH class of DUBs, is one of the most well studied DUBs, and was identified in our prostate cancer protein profiling study [3,5,7]. Although, previous data demonstrate a putative role of UCHL1 in different tumor types, the exact oncogenic mechanism remains unclear. Deregulation of UCHL1 has been observed in solid tumors such as pancreatic cancer [8], non-small cell lung cancer [9], colorectal cancer [10], osteosarcoma [11], and oesophageal cancer [12]. Furthermore, it has been reported that UCHL1 overexpression is associated with tumour progression, size and invasiveness [10]. In gallbladder cancer UCHL1 is overexpressed due to hypomethylation of its promoter and the enhanced activity of the gene correlates with metastasis [13]. In contrary, promoter hypermethylation leading to silencing of UCHL1 has been reported in progression of squamous cell carcinoma as well as gastric cancer and in pancreatic cancer cell lines [14-16]. Recent reports demonstrated that UCHL1 plays a key role in dissemination of non-small cell lung cancer [17] and an association of UCHL1 with β-catenin signaling pathway [18]. Functional genomics studies revealed that siRNA mediated downregulation of UCHL1 regulates expression of several genes which are involved in multiple cellular processes such as apoptosis, cell proliferation and migration [19]. Mutations in the UCHL1 gene have been shown to be associated with Parkinson's disease rather than cancer, for which differential expression appears to be more common. Expression profiling data from various tumour types reported that UCHL1 is either up- or downregulated due to promoter hypo- or hypermethylation depending on the type of malignant tissue. Li et al. showed that UCHL1 promotes tumour suppressor p53 signaling and is silenced due to its promoter methylation in nasopharyngeal carcinoma [20].

In our previous proteomic profiling study, we have identified a list of differentially expressed proteins in cancer containing several proteins that are known to be dysregulated in prostate cancer [3]. Among them we identified UCHL1 as being downregulated in PCa compared to surrounding histological normal tissue or benign prostate epithelium. It has been reported that UCHL1 is deregulated in multiple types of tumours and the precise mechanism of the downregulation and function of UCHL1 in prostate cancer progression have not been investigated before. Therefore, the main objective of the present study was the functional characterization of UCHL1 in prostate cancer progression. The methylation status of UCHL1 promoter in tissue samples and the effect of altered UCHL1 expression on different cellular events were examined to determine the role of UCHL1 expression in PCa. We found that UCHL1 is downregulated in PCa due to promoter hypermethylation and demonstrated that UCHL1 has tumour suppressor activity in LNCaP cells.

## 2 Methods

### Clinical samples, ethics statement and protein extraction

Prostate tissue samples were obtained from the University Medical Center Hamburg Eppendorf after informed consent. The study was approved by the local ethics committee of the University Hospital Eppendorf, Hamburg. For expression profiling whole prostates were collected after radical prostatectomy from patients with elevated PSA values and preoperative pathological examination performed at Martini Clinics, Hamburg, Germany. Patients received no preoperative therapy. After radical prostatectomy samples were frozen in liquid nitrogen until use. Tumor and benign areas were marked on the sections. We employed manual micro dissection method to obtain pathologically characterized materials for gene and protein expression profiling. The corresponding areas on the remaining blocks were sliced out with sharp knife, embedded in Tissue-tek^® ^and stored at -80°C until use for total protein extraction. Protein preparation has been described previously [3].

### RNA and DNA extraction from tissue sections

After surgical removal of the prostate, tissue samples were immediately taken with a 6 mm punch biopsy instrument (Stiefel, Wächtersburg, Germany) from areas that were suspected to contain tumor foci based on information obtained from the preoperative systematic biopsies. Tissue biopsy was immediately immersed in RNAlater (Qiagen, Hilden, Germany), stored overnight at ambient temperature and frozen at -20°C until use. For nucleic acid isolation, the specimen was thawed at room temperature and immediately washed two times each of 5 minutes in 10 ml ice-cold sterile PBS-buffer to elute most of the RNAlater from the tissue. Cryo sections were taken by fixing the tissue using Tissue-Tek^® ^(Sakura, Netherlands) followed by freezing in a cryo-microtome and stained with haematoxylin and eosin (H&E) and analyzed by pathologists. Tissues were only enrolled into the study if at least 70% of cells were epithelial prostate tumor cells. Then, 10-15 subsequent unstained sections were transferred to a cryo tube for RNA and DNA isolation and the final section was again H&E stained and analyzed by pathologists. In parallel, normal prostate tissues were collected from tumor free areas and processed in the same way as tumor samples. Adjacent cryo sections were used for DNA and RNA extraction from the same prostate tissue specimens. Total RNA and DNA were extracted using the All Prep DNA/RNA Mini kit (Qiagen) according to the manufacturer's instructions. The quantity of the DNA and total RNA was checked using the Nanodrop and RNA quality by Bioanalyzer. Samples with low RNA quality (RIN < 6) were excluded from further analysis.

### Cell culture

The PCa cell lines LNCaP and DU145 were purchased from DSMZ (Braunschweig, Germany) and cultivated in RPMI1640 (Invitrogen) supplemented with 10% fetal bovine serum, 100 units/mL) penicillin and streptomycin as recommended by suppliers Phoenix amphotrophic packaging cells were grown in DMEM with 10% fetal bovine serum (FBS) and penicillin/streptomycin. Cells were regularly tested for mycoplasma contamination using the MycoAlert Kit (Cambrex Bio Science Rockland, Inc., Rockland, ME, USA).

### Bisulfite treatment and methylation specific PCR (MSP)

DNA isolation from prostate tissues was performed using Qiagen all prep kit according to supplier's protocol. The DNA concentration was measured by nanodrop spectrophotometer (Peqlab, Germany). Approximately 1 μg DNA was sodium bisulfite-modified and subjected to MSP with primers specifically recognizing the unmethylated or the methylated sequence of UCHL1. MSP primers for the UCHL1 gene were adapted from previous publication [21]. Primers used for MSP are mentioned in additional files (Additional file 1). The PCR was run for 35 cycles with an annealing temperature of 56°C. Normal DNA from peripheral blood was treated in vitro with *Sss*I methyltransferase (New England Biolabs, Beverly, MA) in order to generate *in vitro *methylated DNA (IVD) that served as a positive control for methylated alleles. PCR products were separated on 2.5% agarose gels and visualized by ethidium bromide staining.

### Bisulfite pyrosequencing

For quantitative analysis of regional DNA methylation, pyrosequencing was used. Following PCR amplification of bisulfite-converted DNA using primer sequences (see Additional file 1) the final biotin-labeled PCR products were captured by Streptavidin Sepharose HP (Amersham Biosciences). PCR products bound on the beads were purified and made single-stranded in a Pyrosequencing Vacuum Prep Tool (Pyrosequencing Inc.). The forward sequencing primers were annealed to single-stranded PCR products and pyrosequencing was performed using the PSQ HS 96 Pyrosequencing system (Biotage AB). Quantification of cytosine methylation was performed using the PSQ HS96A 1.2 software package.

### RNA isolation and quantitative real time PCR

Quantitative real time PCR for analysis of transcriptional levels of UCHL1 was performed in 48 benign and 45 tumour samples using SYBR Green. RNA isolation and cDNA synthesis carried out according to standard protocols. Quanti Tect primers for UCHL1, p53, MDM2 and p27Kip1 and GAPDH (housekeeping gene) were purchased directly from Qiagen, Germany. Quantitative real time PCR was performed in thermal cycler (Stratagene, Germany) using Dynamo Flash SYBR Green qPCR kit (Finnzymes, Finland) under optimized cycling conditions. PCRs for the target and housekeeping genes were performed in triplicates and mean relative expression levels were reported. To obtain statistical significance data obtained were analyzed by unpaired student *t*-test and p value < 0.05 was considered as significant. For semiquantitative UCHL1 RTPCR we used the cloning primers and for RPLP0 primers refer additional files (Additional file 1).

### Cloning strategy for UCHL1 overexpression

An UCHL1 protein expressing recombinant vector was generated by cloning the coding region of the human UCHL1 (Accession number NM_004181) cDNA derived from prostate tissue into the pMSCV-Puro vector (Clontech, Palo Alto, CA, USA). UCHL1 coding sequence (CDS) was amplified from total cDNA by PCR using Phusion DNA Polymerase (Finnzymes Oy, Finland). After digestion of the PCR product and pMSCV vector with BglII/XhoI enzymes (Fermentas GmbH, Germany), ligation of the PCR product with linear vector resulted in recombinant pMSCV-UCHL1 construct. The sequence of the cloned PCR fragment was confirmed by DNA sequencing (MWG Operon).

### Virus production and infection of target cells

Phoenix amphotrophic packaging cells were transfected with either empty or pMSCV-UCHL1 vector using CaCl_2 _transfection method. The transfection mixture was prepared by mixing 15 μg of plasmid DNA and 125 mM Cacl_2 _in 1 ml of HBS. The DNA precipitate was added drop wise into the cell culture medium containing 25 μm of chloroquine. After 12 h of transfection, medium was replaced with fresh medium and further incubated for 12 h. The virus containing medium was collected, filtered by 0.45 μm sterile filters directly on the target cells at around 50% of confluence. The cells were fed with fresh medium to continue another round of virus collection. Both target and packaging cells were continued to grow. 12 h later, two more infection cycles were repeated. After three cycles of infection, the target cells were grown in normal cultivation medium for 24 hrs and selected for integration of the target gene and/or puromycin at a concentration of 2 μg/ml until all cells died in control dishes. The colonies appeared with resistance to puromycin were propagated further and verified for overexpression of UCHL1 in LNCaP cells.

### Cell proliferation assay

For cell proliferation assays, cells were plated at a density of 1.5 × 10^5 ^cells/well in 6-well format in complete growth medium. Cells were allowed to grow under optimal culture conditions over a period of 0 to 8 days. Cells were harvested by trypsinization and counted using Vi-Cell Cell counter (Beckaman Coulter GmbH, Germany). The growth rate was shown by plotting the mean total number of cells from triplicate experiments vs. growth time in days. Each experiment was performed in triplicate wells and repeated 3 times. The significance of difference in growth between UCHL1 positive and mock LNCaP cells was calculated using student t-test.

### SA-ß-gal-staining

Detection for SA-ß-galactosidase was performed as described elsewhere [22]. Briefly, LNCaP cells were harvested at sub confluent density and fixed with 2% PFA and 0.25% glutaraldehyde in PBS supplemented with 1 mM MgCl2 (pH 6.0) and incubated in a staining solution containing potassium cyanide/X-gal in PBS/MgCl2 (pH 6.0) at 37°C over night. Slides were analyzed using an Axioplan microscope (10 × magnifications) (Carl Zeiss AG, Germany). 200 cells/triplicate were analysed for a positive staining.

### Colony formation assay

Effect of UCHL1 expression on anchorage-independent LNCaP cell growth was analyzed by soft agar assays. In soft agar assay, bottom agar was prepared by mixing 1% of agarose (Bacto Agar: Becton, Dickinson, Sparks, MD) with 2 × RPMI 1640 with 10% FBS in 6-well plates at 37^°^C to achieve final concentration of 0.5% of agar. After solidifying the bottom agar, 1 × 10^4 ^cells were mixed with cultivation medium and agar solution to obtain a final concentration of 0.35% agar. The mixture was spread on the surface of pre prepared base agar plates immediately. The culture medium was replenished every 3 days with fresh medium. After 14 days of incubation, plates were stained with 0.005% crystal violet solution until colonies turned purple color. After washing excess stain solution colonies were photographed and counted under a light microscope. Each experiment was performed in triplicates and repeated 3 times.

### Proteasomal activity assay

Cells were lysed in M-PER buffer with complete protease inhibitor cocktail without EDTA (Roche, Germany). Protein concentration was measured by BCA method (Thermo Fischer Scientific, Germany) according to supplier's protocol. For proteasomal activity assay, 10 μg of total protein diluted to final volume of 50 μl with incubation buffer (5 mM DTT, 0.5 mMEDTA, 20 mM HEPES, 0.1 mg/ml ovalbumin in ddH_2_O, pH 7.8). Protein lysate prepared in incubation buffer were pre incubated for 2 h at 4°C. For blank control, incubation buffer alone was included in each assay. After incubation, the substrate suc-LLVY-AMC (Calbiochem) was added to the incubation mixtures to achieve final of concentration of 60 μM in final volume of 100 μl. The assay plate was incubated at 37°C for 1 h in the dark before measuring the proteasomal activity in fluorescent spectrophotometer (Mitras LB 940, Berchthold Technology, TN, US) at 355 and 460 nm. Each experiment was performed in triplicates and repeated 3 times.

### Western blotting

Commercially available antibodies against protein targets of interest were purchased. Protein extracts prepared in M-PER (Pierce) with protease and phosphatase inhibitors were separated by 4-12% Bis-Tris-NuPAGE in Nupage running buffer and electrophoretically transferred onto PVDF membrane (Millipore). Blocking was carried out in 1 × Rotiblock solution (Roth Chemicals) followed by incubating the membrane with primary antibodies purchased all from cell signaling except anti UCHL1 from Millipore diluted at 1:1000 in 3% BSA in TBST overnight at 4°C. Excess antibodies were removed by washing with NaCl-Tris-Tween 20. Incubation with secondary antibody conjugated to horseradish peroxidase [anti-(mouse IgG) or anti-(rabbit IgG) from cell signaling, diluted 1:5000 in 1 × Rotiblock] was performed for 1 h at room temperature. After three washes, the reaction was developed by the addition of LumiGLO substrate (Thermo). The emitted light was captured on X-ray film (GE Healthcare).

### Statistics

Cell culture experiments were carried out in triplicates and repeated three times. Data points were expressed as mean of triplicates and median of repeated experiments. Graph Pad PRISM Version 5.0 statistics program was used to test significance of the results with Mann-Whitney test or t-test and p values less than 0.05 with 95% confidence interval were considered as significant.

## 3 Results

### UCHL1 expression and promoter DNA methylation status in PCa patients

**Figure 1:**
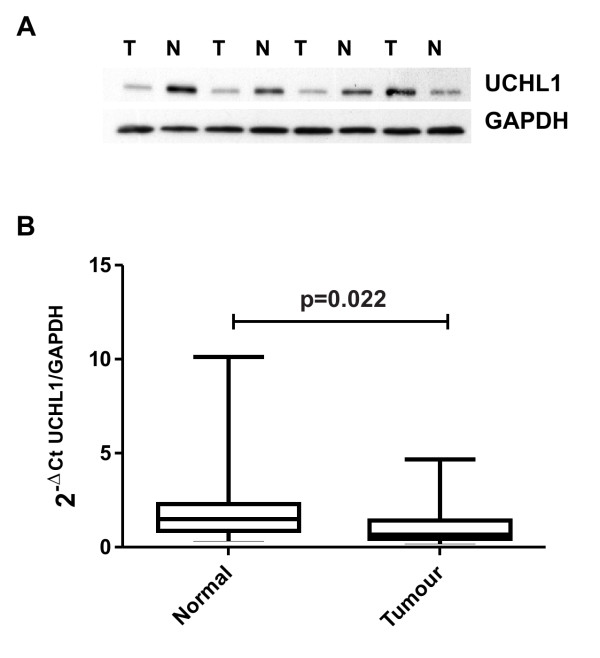
**UCHL1 protein and mRNA expression in prostate cancer**. **(A) **UCHL1 is downregulated in 32 out of 40 PCa patients. A representative Western blot was shown here. GAPDH is used as an internal loading control. **(B) **Quantitative RT-PCR of UCHL1 transcripts from prostate cancer tissue and normal prostate tissues. The ratio of UCHL1 expression was normalized against GAPDH expression and is graphically presented as box plots (t-test was used to analyze statistical significance).

**Figure 2:**
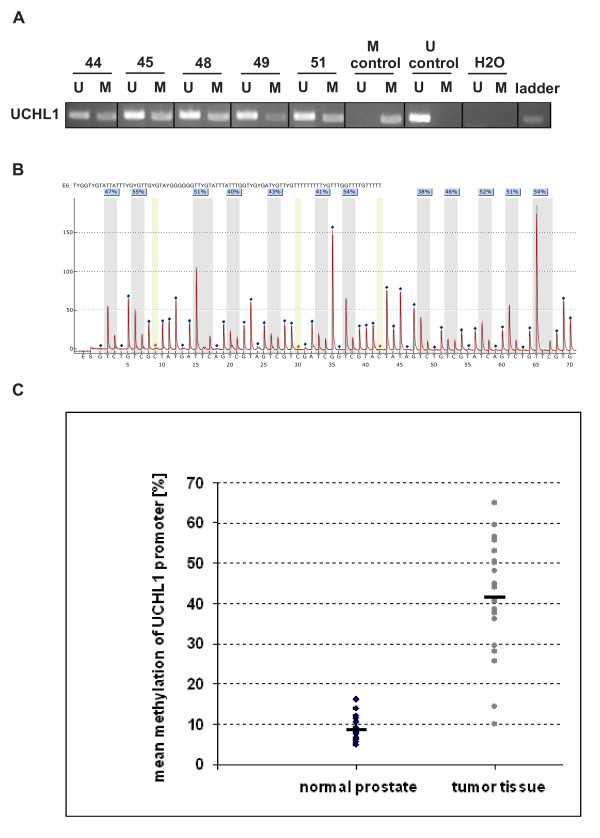
**UCHL1 expression is regulated by promoter methylation in PCa**. **(A) **MSP results show highly (44, 45, 48 and 51) and weakly (49) methylated tumour samples as well as positive (M) and negative (U) controls. The M lane shows amplification with primers specific for methylated CpG sites and the U lane with primers specific for unmethylated CpG sites. The positive control is obtained from in vitro methylated DNA and the negative control from peripheral blood of a healthy donor. **(B) **The pyrogramm of one sample covers 12 CpG sites (grey bars) in the promoter region of UCHL1 with a methylation rate between 35 and 55% (mean 48%). Nucleotides confirming complete bisulfite conversion are shown in yellow bars. **(C) **The pyrosequencing of 20 normal and 20 prostate cancer samples revealed a significant higher rate of methylation for the cancer tissue. 18 tumour samples show a methylation density in the promoter region of UCHL1 clearly higher than the normal prostate tissue.

We have recently demonstrated proteomic profiling on pathologically characterized prostate tissue sections with a list of differentially regulated proteins in tumour compared to surrounding histological normal tissue. Protein profiling revealed significant downregulation of UCHL1 in prostate cancer patients. Further validation of UCHL1 protein expression in a new independent set of samples confirmed that 32 out of 40 patients with lower UCHL1 levels in tumour tissue than in the surrounding benign prostate epithelium. The representative western blot is shown in Figure 1A. In analogy, the measurement of UCHL1 mRNA levels using quantitative real time PCR confirmed significant downregulation of UCHL1 at transcriptional level (Figure 1B) in prostate cancer. Based on the fact that the UCHL1 expression is regulated via promoter methylation in different types of cancer, we investigated the methylation status of the UCHL1 promoter regions in 20 samples of normal prostate tissue and 20 tissue samples of prostate cancer by MSP. The MSP analysis of normal prostate tissue showed 11 samples without methylation signal, 6 with very weak methylation and 2 samples with a weak methylation and 1 sample with a high methylation. Of the 20 prostate cancer tissue samples, 1 was unmethylated, 1 showed a very weak methylation, 3 samples were detected with a weak and 15 samples with a highly methylated promoter region (Figure 2A). Due to the high sensitivity of MSP with a low specificity, we made complementary evaluation of DNA methylation with pyrosequencing. Using pyrosequencing with 2 different sequencing primers, we quantified the methylation of 15 CpG sites in the promoter region of UCHL1 (Figure 2B). The mean methylation of all normal prostate tissue samples was 8.6% (5.5% - 16%). In contrast, the prostate cancer tissue showed a mean methylation of 41.4% (10% - 69%) (Figure 2C). The methylation rate was constant for the 15 CpG sites analyzed without significant differences between single CpG sites. Only 2 prostate cancer tissue samples revealed a methylation below the highest methylation of normal prostate tissue. According to the high sensitivity with a low specificity of MSP, we considered pyrosequencing with quantification of the methylation rate as the method of choice for the distinction between normal and tumour tissue.

### Expression of UCHL1 in PCa cell lines

**Figure 3:**
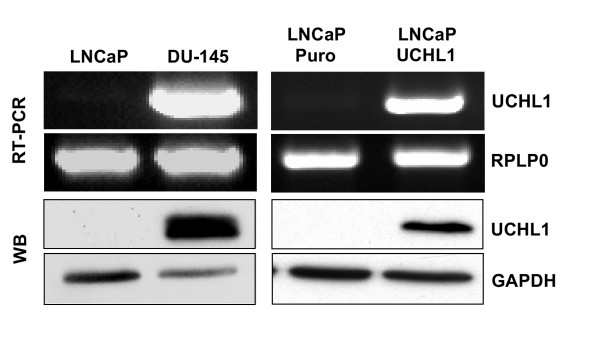
**UCHL1 expression in wild type and stably transfected prostate cancer cell lines**. UCHL1 mRNA (upper panel) and protein (lower panel) are abundantly expressed in DU145, while LNCaP cells show an absence of UCHL1 expression. The housekeeping gene RPLP0 for RT-PCR and GAPDH for Western blotting confirm equivalent loading of samples. Exogenous stable expression of UCHL1 is obtained in androgen dependant prostate cancer cells (LNCaP) by retroviral transduction. For confirmation of expression of UCHL1 mRNA and protein, RT-PCR (upper panel) and Western blotting (lower panel) were performed respectively.

To analyze expression of UCHL1 in PCa cell lines, we performed semi-quantitative RT-PCR and Western blotting on LNCaP and DU145 cells to investigate the role of expression of UCHL1 in prostate cancer initiation and progression. RT-PCR and Western blotting showed abundant expression of both UCHL1 mRNA and protein in DU145 cells, but absent expression in LNCaP cells. As a house keeping gene RPLP0 levels were measured in RTPCR for normalization of cDNA prepared from mRNA and anti-GAPDH antibody were used as a loading control for western blotting (Figure 3). This result is in line with published literature by Leiblich et al. [23] showed that UCHL1 is silenced by promoter methylation in LNCaP cells. Therefore we used LNCaP as model to study function of UCHL1 in PCa development.

To assess the physiological effects of UCHL1 expression on prostate cancer cells, UCHL1 protein producing constructs were generated and the target gene was transferred into LNCaP cells by retroviral transduction. The expression of UCHL1 mRNA and protein in LNCaP cells were verified by RTPCR and western blotting (Figure 3) using an anti-UCHL1 antibody for western blotting. The UCHL1 transduced cells showed clear overexpression of UCHL1 mRNA and protein.

### UCHL1 activity on LNCaP cell proliferation, cellular senescence and anchorage-independent growth

**Figure 4:**
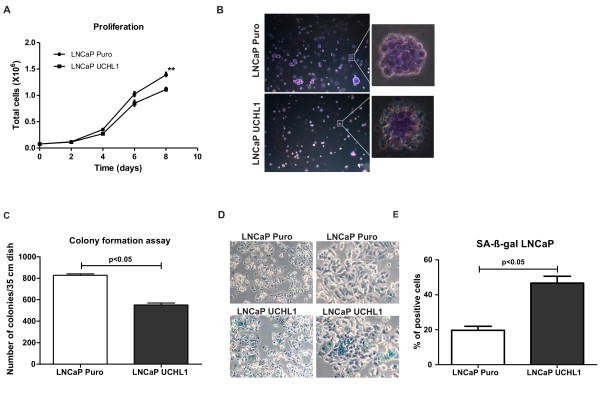
**Influence of UCHL1 overexpression on the phenotype of LNCaP cells**. **(A) **Cell proliferation after overexpression of UCHL1 and cell viability was measured by using Vi-CELL Cell Viability Analyzer. (B) Colony formation assay in soft agar for LNCaP cells with (UCHL1) or without UCHL1 (Puro) expression. UCHL1 positive cells (top) show significant suppression in the number of colonies appearing in soft agar and in the colony morphology compared to mock cells (bottom). **(C) **After crystal violet staining the number of colonies appearing on soft agar plates was counted using an invert microscope and the results are expressed as mean ± SD of 3 independent experiments (*p *< 0.005). **(D) **Senescence was quantified using SA-ß-gal-staining. **(E) **Number of SA-ß-gal positive cells was counted using an Axioplan microscope and the results are depicted as mean ± SD of 3 independent experiments.

Since downregulation of UCHL1 is a prominent feature of primary prostate cancer cells, we assumed a crucial role for UCHL1 as a potential tumour suppressor. Indeed, exogenous overexpression of UCHL1 in LNCaP cells had a significant impact on in vitro growth capacity (Figure 4A) and reduced proliferation of these cells compared to corresponding mock transduced control cells. More strikingly, UCHL1 overexpression suppressed anchorage-independent proliferation in a soft agar assay (Figure 4B-C). Whereas LNCaP cells transduced with the mock vector only gave rise to numerous colonies, UCHL1 overexpression significantly suppressed anchorage-independent growth and reduces the transformed phenotype of LNCaP cells. To unveil the putative mechanisms that impacted on proliferation and transformation capacity, we investigated apoptosis and senescence in UCHL1 overexpressing cells. An increased rate of apoptosis could not be observed (data not shown). Therefore, we assessed the endogenous level of ß-galactosidase as an indicator for senescence in LNCaP UCHL1 expressing cells. Indeed, we could observe more cells with an increased level of SA-ß-gal in cells overexpressing UCHL1 compared to the respective mock control cells, suggesting that the overexpression of UCHL1 leads to the induction of cellular senescence in LNCaP prostate cancer cells (Figure 4D-E). These results support the hypothesis of UCHL1 as a tumour suppressor in the pathogenesis of prostate cancer, in parts by inducing senescence as a permanent cell cycle arrest.

### UCHL1 overexpression leads to K63 specific de ubiquitination in LNCaP cells

**Figure 5:**
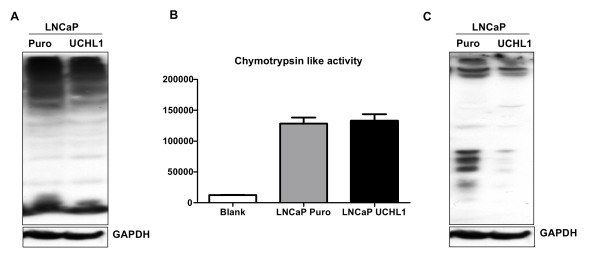
**In LNCaP cells exogenous expression of UCHL1 influences K63 ubiquitylation**. **(A) **Representative Western blots against mono- and polyubiquitin from LNCaP cells stably expressing UCHL1 or empty vector as control. GAPDH is used as an internal housekeeping protein to ensure equal loading of samples. **(B) **Measurement of chymotrypsin like activity of the proteasome in UCHL1 positive or mock LNCaP cells served as control. The results are expressed as the mean ± SD from 3 independent experiments each in triplicates. **(C) **UCHL1 overexpression in LNCaP cells reduces the level of K63 chain specific ubiquitylated proteins detected by Western blotting with anti K63 linked polyubiquitin antibody. GAPDH is used as loading control.

In order to evaluate, whether UCHL1 activity regulates addition and/or removal of ubiquitin to proteins in LNCaP cells, ubiquitination of proteins in UCHL1 positive LNCaP and LNCaP puro cells was tested with anti-Ubiquitin antibodies. Stable overexpression of UCHL1 in LNCaP cells did not show any change in the pattern of ubiquitination of proteins by Western blot (Figure 5A). Furthermore, to investigate the role of UCHL1 on proteasomal activity; we measured chymotrypsin-like activity in UCHL1 positive LNCaP and mock control cells. Chymotrypsin-like activity did not significantly change with overexpression of UCHL1 in LNCaP cells (Figure 5B). These data indicate that UCHL1 does not alter ubiquitin levels and proteasomal activity in LNCaP under baseline conditions. In ubiquitin-proteasome pathway, ubiquitin molecules are linked together in chains to a protein, are covalently coupled via an isopeptide bond utilizing the lysine residues of each ubiquitin. Substrate proteins and are linked to ubiquitin using distinct ubiquitin lysine residues at 6, 11, 29, 48 and 63 positions in ubiquitin and influence distinct cellular events. K48-linked polyubiquitin chains mainly target proteins for proteasomal degradation, while K63-linked polyubiquitin regulates protein function, sub cellular localization, or protein-protein interactions. UCHL1 is described to possess K63 ligase activity in vitro upon dimerization. Therefore we used anti- lysine 63-linkage (K63) specific antibodies, to see whether UCHL1 had any influence on K63 ubiquitin linkage in LNCaP cells. Interestingly, Western blot results showed consistent loss of K63 ubiquitylated proteins (Figure 5C). These results together indicate that in LNCaP cells UCHL1 activity may be associated with regulation of localization, function and interaction of proteins to control various cellular events, rather than regulation of ubiquitin levels.

### UCHL1 suppress anchorage- independent growth and cell proliferation via AKT phosphorylation and stabilizing p53 levels

**Figure 6:**
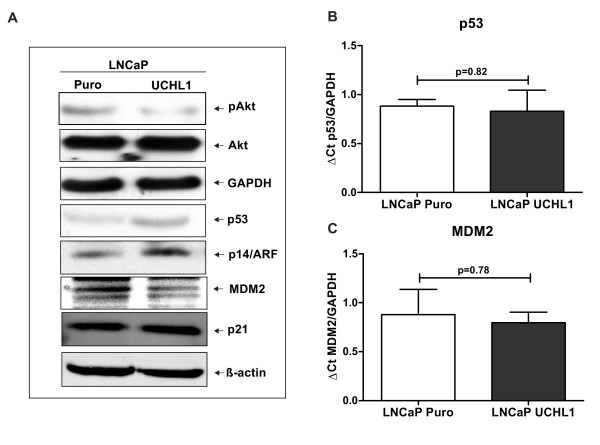
**UCHL1 suppresses anchorage-independent growth and cell proliferation via AKT phosphorylation and stabilization of p53 levels**. **(A) **Representative Western blots showing decreased levels of phospho AKT in UCHL1 positive LNCaP cells. Furthermore, accumulation of p53, decreased MDM2 levels and an increase in p14ARF without any influence on p21 was shown by Western blot. β-Actin was used as controls to ensure equal loading of samples. **(B-C) **Quantitative RT-PCR revealed no changes of p53 and MDM2 mRNA levels in UCHL1 positive cells. The results are indicated as mean ± SD of 3 independent experiments.

We therefore further investigated how UCHL1 regulates LNCaP cell growth in-vitro. As we observed in soft agar assays, UCHL1 significantly suppressed anchorage independent growth. The ability of anchorage independent growth of tumour cells has been linked to the PI3 kinase/AKT pathway and is associated with the metastatic potential of cancer cells [24]. Our results show significant reduction in the phosphorylation of AKT at S473 in UCHL1 positive LNCaP cells compared to the mock control cell line (Figure 6A). However, the key mechanism involved in UCHL1 mediated growth suppression needs to be investigated. p53 is an upstream regulator of the PI3 kinase/AKT pathway via PTEN in various cancers. Western blots again indicated that UCHL1 overexpression in LNCaP cells induces accumulation of p53 whereas MDM2 protein is decreased compared to mock control cell line (Figure 6A). This observation is not correlated positively with real time PCR results showing no significant changes in p53 (*P-value *= 0.82) and MDM2 (*P-value *= 0.78) mRNA levels in UCHL1 positive cells (Figure 6B-C). Together, these results indicated that the UCHL1 play a key role in regulation of stability of p53 and MDM2 by deubiquitination but not at transcriptional level expression. Interestingly, from the known p53 network, p14ARF is also significantly upregulated in UCHL1 positive cells. These results are consistent with the previous findings that the tumour suppressor p14ARF inhibits p53 degradation via inhibition of E3 ligase activity of MDM2 which directs MDM2 for degradation by proteasome. As reported previously, increase in p53 protein levels in UCHL1 positive LNCaP cells may regulate cyclin dependant kinase inhibitor p21. Exogenous expression of UCHL1 in LNCaP cells showed no effect on the expression of p21, which indicates that the regulation of p53 has no effect on p21 stability (Figure 6A). Taken together, it appears that UCHL1 suppresses the growth of LNCaP cells via stabilization of tumour suppressor protein such as p53 and by inactivating AKT/PKB pathway.

### Exogenous expression of UCHL1 results in accumulation of p27Kip1 and suppressed Cyclin A expression

**Figure 7:**
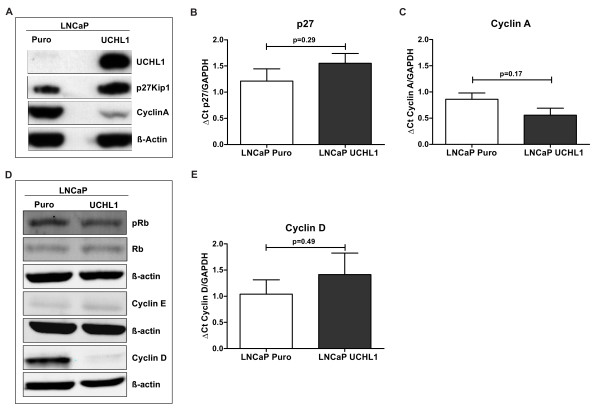
**Effects of overexpression of UCHL1 on the expression of cell cycle related proteins**. **(A) **Representative Western blots against UCHL1, p27Kip1, Cyclin A and β-actin, as a house keeping protein, demonstrated that UCHL1 induces accumulation of p27Kip1 and reduction of Cyclin A expression in LNCaP cells. **(B-C) **Quantitative RTPCR revealed no statistically significant change of p27Kip1 and Cyclin A mRNA level in UCHL1 positive cells. The results are indicated as mean ± SD of 3 independent experiments. **(D) **Protein levels of Rb, pRb, Cyclin E, Cyclin D and β-actin were detected by Western blotting analyses using corresponding antibodies in UCHL1 overexpressing LNCaP cells. **(E) **Quantitative RTPCR revealed no statistically significant change of Cyclin D mRNA level in UCHL1 positive cells. The results are indicated as mean ± SD of 3 independent experiments.

Previously it has been shown that UCHL1 interacts with p27Kip1, a cyclin dependant kinase inhibitor. P27Kip1 mediates cell cycle arrest at G1 results in senescence of Cells. To understand whether LNCaP growth arrest and senescence was mediated over an effect of UCHL1 on p27Kip1 stability, we have measured p27Kip1 levels using anti- p27Kip1 antibody in UCHL1 positive cells. Immunoblotting with whole cell lysate revealed that p27Kip1 protein levels were significantly elevated in consequence of UCHL1 overexpression in LNCaP prostate cancer cells lines where as the amount of p27Kip1 mRNA was not significantly correlated with protein levels (Figure 7A-B). To investigate the consequences of elevated p27Kip1 by UCHL1 protein in LNCaP cells, we extended our analysis by measuring cell cycle proteins. As described before, p27Kip1 blocks Cyclin E dependant transactivation of Cyclin A. We have measured the expression of Cyclin A in UCHL1 positive cells and our results clearly demonstrate that elevated levels of p27Kip1 in LNCaP cells due to overexpression of UCHL1 significantly reduced Cyclin A expression on protein level (Figure 7A). mRNA was not significantly changed in UCHL1 expression LNCaP cells (Figure 7C).

To understand the impact of UCHL1 accumulation on cell cycle control of LNCaP cells, we examined the expression and phosphorylation of retinoblastoma proteins (Rb) predominantly regulated by p21 as well as Cyclin E and Cyclin D1 proteins associated with p27Kip1. Notably, we found no change in levels of either total or phosphorylated Rb in cells with a p27Kip1 accumulation. Interestingly, Cyclin D1 which promotes Cyclin A expression is significantly reduced on protein level due to high p27Kip1 activity in UCHL1 overexpressing LNCaP cells where as Cyclin E remains unchanged (Figure 7D). In line with Cyclin A the mRNA of Cyclin D was not significantly regulated in UCHL1 overexpressing cells. (Figure 7E).

These results together suggest that the accumulation of p27Kip1 as a result of UCHL1 overexpression might interfere with cell cycle progression and thus influence cell growth.

## 4 Discussion

### UCHL1 downregulation in PCa is associated with epigenetic modifications

In the present study, we analyzed the mechanism of UCHL1 downregulation in PCa and the role of UCHL1 as tumour suppressor in LNCaP prostate cancer cells. Our proteomic analysis of prostate tissue samples highlighted significant downregulation of UCHL1 in cancer compared to the surrounding benign tissue [3]. In the current study, we analyzed whether downregulation of UCHL1 in PCa is due to promoter hypermethylation. Methylation specific PCR and pyrosequencing results demonstrated that UCHL1 suppression in primary prostate tumour tissues is linked to its promoter DNA methylation. This hypermethylation is correlated with low UCHL1 transcripts and protein levels in cancer compared to adjacent benign prostate epithelium.

### UCHL1 has tumour suppressor role PCa via p53 accumulation

Our results have shown that UCHL1 inhibited LNCaP cell growth. Furthermore, UCHL1 significantly suppressed anchorage independent growth in soft agar. In cancer cells, anchorage independent growth and the metastatic potential have been linked to the AKT/PI3 kinase pathway [24], that the tumor suppressor p53 as an upstream regulator of AKT/PI3 kinase pathway directly suppress PTEN a tumour suppressor and negative regulator of AKT pathway [25-27]. In our results we found that there was a significant increase in p53 in UCHL1 positive cells, but no influence of UCHL1 on PTEN expression (data not shown). It is therefore intriguing to speculate that the observed suppression of tumorigenesis by UCHL1 is due an effect of p53 on activated Akt/PI3 kinase pathway regardless of PTEN expression. In p53 signaling pathway, the tumor suppressor p14ARF inhibits the E3 ligase MDM2 activity, which in turn inhibits p53 degradation. Furthermore p53 can act as a transcription factor attenuates MDM2 function by suppressing transcription of MDM2 [28,29]. Our results also show that p14ARF is upregulated in UCHL1 positive cells possibly explaining the observed suppression of MDM expression.

Therefore we would speculate that UCHL1 controls p53 activity indirectly via multiple mechanisms. Together, these finding are in line with the results of Tokumaru et al. describing tumor suppressor function of UCHL1 in head and neck squamous cell carcinoma [30]. The cyclin dependent kinase inhibitor p21 is the major transcriptional target of p53 and is required for p53 dependant cell cycle arrest [31-33]. p21 regulates cell cycle progression at the S phase by direct inhibition of cyclin E/CDK2 and cyclin D/CDK4 complexes activity. We could not demonstrate an influence of p53 on the expression of p21 in UCHL1 positive LNCaP cells. We therefore suppose that the accumulated p53 targets AKT phosphorylation and thereby cell survival, rather than p21 in LNCaP cells. Consistent with our observation that p53 and p14ARF are upregulated in UCHL1 overexpressing cells, we detected a p27Kip1 upregulation and a senescent phenotype by using the SA-ß-gal assay. The importance of p27kip1 stabilisation for induction of a senescent phenotype in murine prostatic intraepithelial neoplasia has been shown by Majumder et al [34]. Furthermore, p53 seems to be involved in radiation-induced senescence [35]. Therefore, we propose that UCHL1 might represent a regulator of senescence induction in prostate carcinogenesis.

### UCHL1 exhibits influence on cell cycle regulator proteins via p27Kip1

UCHL1 may be involved in multiple cellular processes. In lung cancer cells, UCHL1 interacts with p27Kip1 [36]. There is an inverse relationship between UCHL1 and p27Kip1 expression in many cell lines [37]. p27Kip1 is a multifunctional protein involved in regulation of cell proliferation and apoptosis [38]. In our hands we observed that UCHL1 overexpression positively regulates p27Kip1 levels in LNCaP cells. However, the precise role of UCHL1 in regulation of p27Kip1 is not well described so far. Earlier reports showed evidence that p27Kip1 is degraded through the ubiquitin-proteasome pathway resulting in decreased cellular concentration [39]. Caballero et al. demonstrated that UCHL1 might be involved in nuclear localization of p27Kip1 mediated via JAB1 in lung cancer cells [36].

To get more insights about the role of UCHL1 via p27Kip1 accumulation, we investigated the mechanism by which proliferation and anchorage-independent growth is suppressed by the UCHL1 in LNCaP. To inhibit cell cycle progression, p27Kip1 needs to be located in the nucleus and interacts with cyclin dependant kinases (CDKs) and/or cyclins to block their activity. As a result, activation of Rb by phosphorylation is blocked and the Rb protein binds to E2F resulting in a positive regulation of cell cycle progression. Guandagno et al. have shown that the expression of cyclin A is critical for anchorage-dependent progression of the cell cycle in NIH3T3 and NRK normal rodent fibroblast cell lines and exogenous expression of cyclin A induced anchorage-independent growth in NRK cells [40]. These findings are consistent with our result, indicating that the downregulation of Cyclin A is correlated with the suppression of anchorage-independent growth of LNCaP cells by UCHL1.

It has also been reported that p27Kip1 inhibits the expression of Cyclin A. p27Kip1^-/- ^knockout in mice is associated with increased cell proliferation due to deficient Cyclin D inhibition [41]. Consistent with these findings we could also demonstrate downregulation of Cyclin D1 in UCHL1 positive LNCaP cells. One of the best known substrates of cyclin D is the Rb tumor suppressor protein. Phosphorylation of Rb is performed by Cyclin D/Cyclin E and Rb remains phosphorylated throughout S, G2 and M phases during cell cycle progression. Zhu et al have shown that phosphorylation of the Rb protein is anchorage-dependent in NIH3T3 and human fibroblast cells. However, in the same report they have also shown convincingly Rb independent regulation of Cyclin A expression in NRK suspension cultures [42]. In our findings we observed that both the expression of Rb or phosphorylation of Rb and Cyclin E expression were not affected by UCHL1 expression in LNCaP cells. Therefore we assume that downregulation of cyclin A in p27Kip1 accumulated LNCaP cells by UCHL1 overexpression is caused by an Rb-independent pathway.

In summary, we showed that *UCHL1 *is suppressed in prostate cancer patients by promoter hypermethylation. Restoration of UCHL1 in LNCap cells in which *UCHL1 *silenced by its promoter methylation could activate p53 signaling axis via reduced AKT phosphorylation and accumulation of p27 kip1 cell cycle inhibitor there by suppressing cellular growth (Figure 4). Thus, our study supplement substantially the current knowledge of the tumor suppressor functions of UCHL1 in cancer progression and postulate that *UCHL1 *hypermethylation could be a potential molecular marker for prostate cancer need to be evaluated in large number of patient cohort.

## List of abbreviations

(UCHL1): Ubiquitin Carboxyl-Terminal Hydrolase 1; (PCa): prostate cancer; (DUBs): deubiquitinases

## Competing interests

The authors declare that they have no competing interests.

## Authors' contributions

RU, EJ, MB, FL, TB, FM, and TS carried out the experiments and analyzed results as well as took part in writing the manuscript. RU, CMS, TS, GS, HS, CB, THB and SB conceived of the study, and participated in its design and coordination and helped to draft the manuscript. All authors have read and approved the final draft of the manuscript

## Supplementary Material

Additional file 1 **Primer sequences used in PCR reactions for the current study**. List of all primer sequences used in MSP, Pyrosequencing for methylation status of UCHL1 promoter, RT PCR and cloning PCR for quantification and amplification of UCHL1 CDS from cDNA respectively. Click here for file
