# Supplementary material for: Ubiquitin carboxyl-terminal hydrolase 1 (UCHL1) is a potential tumour suppressor in prostate cancer and is frequently silenced by promoter methylation
Source: Mol Cancer. 2011 Oct 14;10:129. doi: 10.1186/1476-4598-10-129 (PMC3212821; doi:10.1186/1476-4598-10-129)
Supplement: Additional file 1 — Primer sequences used in PCR reactions for the current study. List of all primer sequences used in MSP, Pyrosequencing for methylation status of UCHL1 promoter, RT PCR and cloning PCR for quantification and amplification of UCHL1 CDS from cDNA respectively. [file 1476-4598-10-129-S1.DOCX]

**Supplementary information**

**Primer sequences:**

1. Primers used for methylation specific PCR

methylated sequence

UCHL1-MF: 5’-TCG TAT TTA TTT GGT CGC GATC-3’

UCHL1-MR: 5’-CTA TAA AAC GCC GAC CAA ACG-3’

unmethylated sequence

UCHL1 UF: 5’-GGT TTG TAT TTA TTT GGT TGT GAT T-3’

UCHL1 UR: 5’-CAA CTA TAA AAC ACC AAC CAA ACA-3’

1. Primers for Bisulfite Pyrosequencing

Forward: 5’-AGT GAG ATT GTA AGG TTT GGG GGT T-3’

Reverse: 5’-bio-ACC YCC CAA ACT ACA ACT ATA AAA C-3’

The forward sequencing primers: 5’-TGT AAG GTT TGG GGG T-3’

5’-GGG GGT TYG TAT TTA TTT G-3’

1. Primers for RT PCR

RPLP0 For: 5'- ttg tgt tca cca agg agg ac -3'

RPLP0 Rev: 5'- gac tct tcc ttg gct tca ac -3'

1. Cloning primers for UCHL1

Forward: 5’-CTAGAGATCTGCCACCATGCAGCTGAAGCCGATGGA-3’ contains a recognition site (underlined) for BglII.

Reverse primer: 5’-CTAGCTCGAGTTAAGCTGCTTTGCAGAGAG-3’ contains a site for XhoI (underlined).
